# Comparative methylomics and chromatin accessibility analysis in ***Osmanthus fragrans*** uncovers regulation of genic transcription and mechanisms of key floral scent production

**DOI:** 10.1093/hr/uhac096

**Published:** 2022-04-22

**Authors:** Yuanji Han, Miaomiao Lu, Shumin Yue, Ke Li, Meifang Dong, Luxian Liu, Hongyun Wang, Fude Shang

**Affiliations:** State Key Laboratory of Crop Stress Adaptation and Improvement, Laboratory of Plant Germplasm and Genetic Engineering, School of Life Sciences, Henan University, Kaifeng, Henan 475004, China; State Key Laboratory of Crop Stress Adaptation and Improvement, Laboratory of Plant Germplasm and Genetic Engineering, School of Life Sciences, Henan University, Kaifeng, Henan 475004, China; State Key Laboratory of Crop Stress Adaptation and Improvement, Laboratory of Plant Germplasm and Genetic Engineering, School of Life Sciences, Henan University, Kaifeng, Henan 475004, China; State Key Laboratory of Crop Stress Adaptation and Improvement, Laboratory of Plant Germplasm and Genetic Engineering, School of Life Sciences, Henan University, Kaifeng, Henan 475004, China; State Key Laboratory of Crop Stress Adaptation and Improvement, Laboratory of Plant Germplasm and Genetic Engineering, School of Life Sciences, Henan University, Kaifeng, Henan 475004, China; State Key Laboratory of Crop Stress Adaptation and Improvement, Laboratory of Plant Germplasm and Genetic Engineering, School of Life Sciences, Henan University, Kaifeng, Henan 475004, China; State Key Laboratory of Crop Stress Adaptation and Improvement, Laboratory of Plant Germplasm and Genetic Engineering, School of Life Sciences, Henan University, Kaifeng, Henan 475004, China; State Key Laboratory of Crop Stress Adaptation and Improvement, Laboratory of Plant Germplasm and Genetic Engineering, School of Life Sciences, Henan University, Kaifeng, Henan 475004, China; Henan Engineering Research Center for Osmanthus Germplasm Innovation and Resource Utilization, Henan Agricultural University, Zhengzhou, Henan 450002, China

## Abstract

Linalool and ionone are two important aromatic components in sweet osmanthus petals, and the regulatory mechanisms that produce these two components remain unclear. In this study, we employed whole-genome methylation sequencing and ATAC-seq technology to analyze the genomic DNA methylation status and chromatin accessibility of the sweet osmanthus cultivars ‘Zaohuang’ and ‘Chenghong Dangui’. Results showed that the promoter region of *TPS2*, a key gene in the linalool synthesis pathway, was less methylated in ‘Chenghong Dangui’ than in ‘Zaohuang’. The chromatin was more accessible in ‘Chenghong Dangui’ than in ‘Zaohuang’, which resulted in a much stronger expression of this gene in ‘Chenghong Dangui’ than in ‘Zaohuang’. This eventually led to a high quantity of linalool and its oxides in the petals of ‘Chenghong Dangui’, but there were lower levels present in the petals of ‘Zaohuang’. These results suggest that DNA methylation and chromatin accessibility play major roles in linalool synthesis in sweet osmanthus. The methylation level of the promoter region of *CCD4*, a key gene for ionone synthesis, was higher in ‘Zaohuang’ than in ‘Chenghong Dangui’. The chromatin accessibility was lower in ‘Zaohuang’ than in ‘Chenghong Dangui’, although the expression of this gene was significantly higher in ‘Zaohuang’ than in ‘Chenghong Dangui’. ChIP-seq analysis and a series of experiments showed that the differential expression of *CCD4* and *CCD1* in the two cultivars may predominantly be the result of regulation by ERF2 and other transcription factors. However, a 183-bp deletion involving the *CCD4* promoter region in ‘Chenghong Dangui’ may be the main reason for the low expression of this gene in its petals. This study provides an important theoretical basis for improving selective breeding of key floral fragrance components in sweet osmanthus.

## Introduction

Sweet osmanthus (*Osmanthus fragrans* Lour.) is a famous aromatic plant whose petals contain >30 kinds of aromatic components, including the monoterpene aromatic substances ionone (such as α-ionone and β-ionone), linalool, and its oxides, which are the main components of the essential oil of sweet osmanthus petals. Essential oils from some Albus group cultivars contain more β-ionone, and certain Aurantiacus group cultivars contain more linalool and its oxides [1–5]. β-ionone and linalool are important aromatic components and represent important fragrances on the commercial market, being widely used and in high demand for the food, cosmetic, and pharmaceutical industries. Therefore, it is important to study the mechanisms of production and regulation of β-ionone and linalool using sweet osmanthus petals as experimental materials for the utilization of these important floral fragrance components and for selective breeding of floral fragrances.

Carotenoid cleavage dioxygenases (CCDs) cleave carotenoids to produce sesquiterpenoid aromatic substances such as ionone, abscisic acid, and other plant growth regulators [6–8]. Studies have shown that the sweet osmanthus carotenoid cleavage dioxygenase OfCCD1 can cleave α-carotene to produce α-ionone and β-ionone and cleave β-carotene to produce β-ionone [9, 10]. The sweet osmanthus OfCCD4 protein cleaves β-carotene to produce β-ionone [8, 11]. Our previous results showed that the sweet osmanthus transcription factors WRKY1 and ERF61 regulate the expression of the sweet osmanthus *OfCCD4* gene and affect the cleavage of carotenoids and the production of β-ionone in petals [4, 12]. Sweet osmanthus *OfCCD1* and *OfCCD4* are two important genes that regulate carotenoid cleavage and α-ionone and β-ionone production. Under high gene expression levels, carotenoids are cleaved in petals, flower color is lighter (light yellow), and ionone content is higher; under low gene expression levels, carotenoids accumulate in petals, flower color is darker (orange-red), and ionone content is lower [11, 13, 14].

Studies have shown that plant *TPS* genes are extensively involved in the production of plant terpenoid metabolites. To date, TPSs have been identified and characterized in many plants, including *Arabidopsis thaliana* [15], *Medicago truncatula* [16], *Antirrhinum majus* [17, 18], *Actinidia* species [19, 20], and *Vitis vinifera* [21]. Genome and transcriptome analysis demonstrated a direct connection between TPSs and abundant aromatic molecules in sweet osmanthus, and *TPS* gene expression also coincided with the production of linalool [3]. Studies have shown that transient transformation of tobacco leaves with *TPS1* and *TPS2* from sweet osmanthus can induce the production of linalool in tobacco leaves, suggesting that TPS1 and TPS2 are involved in the production of linalool [2].

Chromatin accessibility is an important determinant of gene expression regulation, and the open state of plant genomic chromatin determines the regulatory network of gene expression during important biological processes such as cell differentiation, growth, and development [22]. Assay for Transposase-Accessible Chromatin using sequencing (ATAC-seq), an innovative technique for studying chromatin accessibility, allows access to all open regions of chromatin in chromosomes under a particular spatiotemporal condition and is an important tool for studying the regulation of gene expression [23, 24]. Sijacic et al. used ATAC-seq to analyze differences in chromatin accessibility between stem cells of *A. thaliana* root tip meristems and differentiated chloroplasts and to construct a regulatory network of transcription factors for these two types of cell development [25].

DNA methylation is an important epigenetic modification that regulates many crucial biological processes. DNA methylation of gene promoter regions can inhibit gene expression and transcription factor binding to promoters [26, 27]. Reduced DNA methylation levels in *A. thaliana* lead to abnormal plant development [28]. DNA methylation levels affect the expression of genes related to papaya flower organ development and inhibit tomato fruit ripening [29].

In this study, we used chromatin accessibility, DNA methylation, and transcription factor regulation to study the regulatory mechanisms involved in the production of β-ionone and linalool and their oxides in sweet osmanthus petals. The results enrich our basic theoretical understanding of the molecular biology surrounding sweet osmanthus and provide a basis for the exploitation of the main floral fragrance components of sweet osmanthus, ionone and linalool and their oxides. They also provide a theoretical basis for the selective breeding of floral fragrance of sweet osmanthus cultivars.

## Results

### Gas chromatography–mass spectrometry (GC–MS) analysis of sweet osmanthus petals

**Figure 1:**
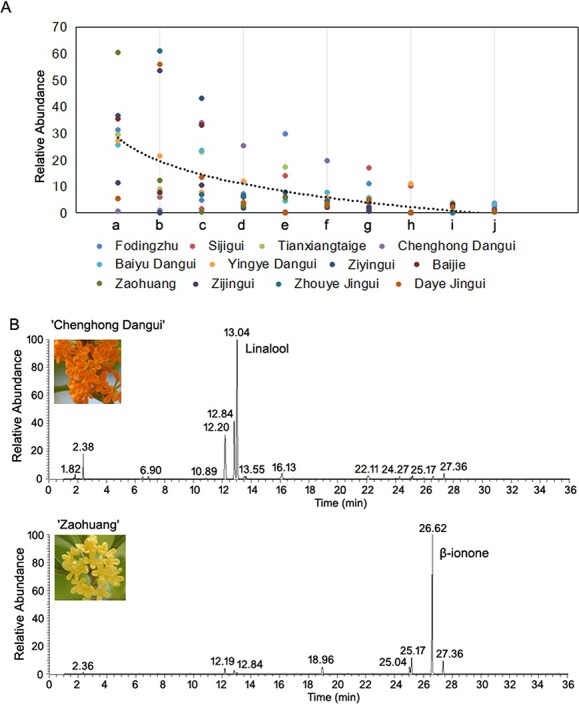
GC–MS analysis of sweet osmanthus petals. A, Analysis of aromatic components in the petals of 12 cultivars. a, β-ionone; b, α-ionone; c, linalool 3,7-dimethyl-1,6-octadien-3-ol; d, trans-5-ethenyltetrahydrahydro-α,α,5-trimethyl-2-furanmethanol; e, dihydro-β-ionone; f, cis-5-ethenyltetrahydrahydro-α,α,5-trimethyl-2-furanmethanol; g, 2(3H)-furanone,5-hexyldihydro-; h, geraniol (2E)-3,7-dimethyl-2,6-octadien-1-ol; i, chloroform; j, 2h-pyran-3-ol,6-ethenyltetrahydro-2,2,6-trimethyl-. B, GC–MS fingerprints of ‘Chenghong Dangui’ and ‘Zaohuang’ cultivars.

To explore the main components of floral aromas and their differences among the four cultivar groups of sweet osmanthus species, we used GC–MS to analyze the aromatic properties of petals from three cultivars of the *O. fragrans* Albus group, three cultivars of the *O. fragrans* Luteus group, three cultivars of the *O. fragrans* Aurantiacus group, and three cultivars of the Asiatic group at their peak of flowering. The results revealed that the monoterpene aromatic components ionone and linalool and its oxides were the main components of the petal essential oil in 12 sweet osmanthus cultivars (Fig. 1A, Supplementary Table 1).

The highest content of linalool and its oxides was found in ‘Chenghong Dangui’ petals, with trans-linalool, cis-linalool, and linalool accounting for 19.58%, 25.26%, and 33.95%, respectively; α-ionone and β-ionone had the lowest contents, accounting for 0.98% and 0.61%, respectively. The lowest content of linalool, and its oxides, trans-linalool and cis-linalool, was found in ‘Zaohuang’, accounting for 2.90%, 2.03%, and 0.88%, respectively, whereas the highest content of β-ionone accounted for 60.58% and the content of α-ionone accounted for 12.28% (Fig. 1B, Supplementary Table 1). Based on the results of GC–MS analysis, we selected two species with large differences in the contents of linalool and its oxides and ionone, ‘Chenghong Dangui’ (D) and ‘Zaohuang’ (Y), as experimental materials for an in-depth study of the mechanisms of production and regulation of the two main aromatic components in flower petals.

### The DNA methylome of petals from the sweet osmanthus cultivars ‘Zaohuang’ and ‘Chenghong Dangui’

Methylome analysis was performed using sulfite sequencing to generate single-base resolution maps of DNA methylation for flower petals of ‘Zaohuang’ and ‘Chenghong Dangui’. Because of the close genetic relationship between the sweet osmanthus cultivars, we used the genome of the sweet osmanthus cultivar “Rixiang Gui” as a reference for analysis [30]. The genome size of the *O. fragrans* Asiatic group cultivar ‘Rixiang Gui’ was 727 Mbp (2n = 46). For each sequencing library, at least 104 M paired-end reads (read length = 150 bp) were produced, generating more than 48 M aligned pairs and more than 39 M unique pairs. For each bisulfite-treated library, >85% of the CG loci were covered, with a conversion rate of >99.3% (Supplementary Tables 2–3). Each methylome-sequencing sample had an average coverage >10-fold per DNA strand. The sequencing coverage and depth were comparable to those of published methylomes of *Arabidopsis*, tomato, and strawberry [29, 31, 32]. Correlation analysis revealed consistency between biological duplicate samples of ‘Zaohuang’ and ‘Chenghong Dangui’ (Supplementary Fig. 1). The mean methylation ratios of CG, CHG, and CHH in the ‘Chenghong Dangui’ genome were 71.55%, 42.29%, and 7.79%, respectively, whereas the mean methylation ratios of the ‘Zaohuang’ genome were 72%, 42.24%, and 7.47% (Supplementary Table 3). The differences in CG and CHG methylation levels between the ‘Chenghong Dangui’ and ‘Zaohuang’ genomes were not significant (*P* > 0.05), but CHH methylation levels were significantly higher in the ‘Chenghong Dangui’ genome (*P* < 0.05，*P* = 0.02).

**Figure 2:**
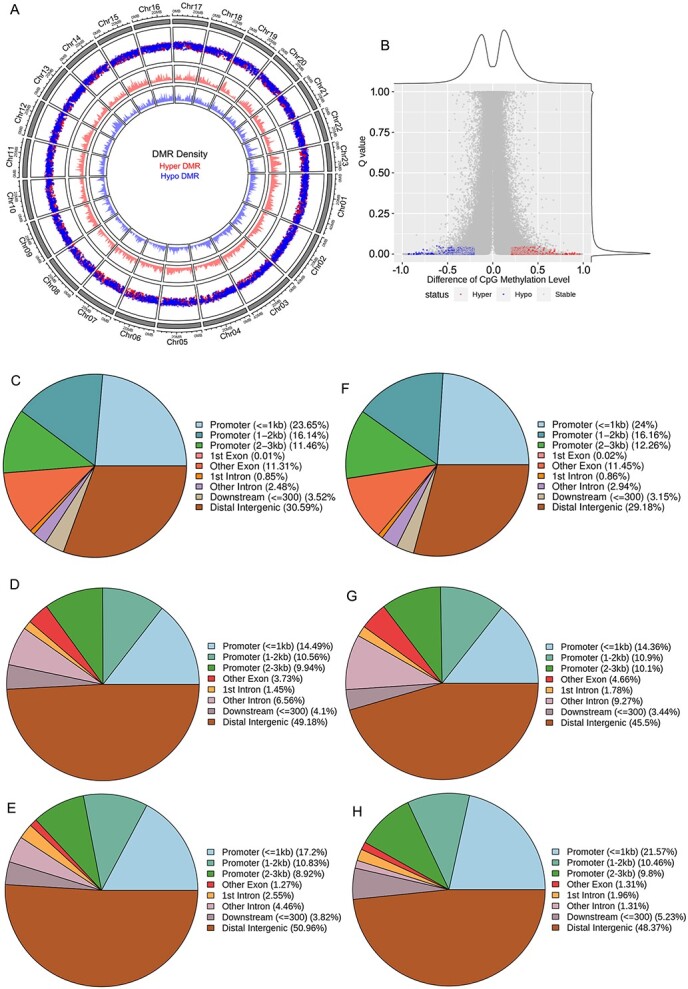
DNA methylation analysis of sweet osmanthus ‘Zaohuang’ and ‘Chenghong Dangui’ petals. A, Distribution of DMRs on 23 chromosomes. Hyper DMR indicates that the methylation level is higher in ‘Zaohuang’ than in ‘Chenghong Dangui’; Hypo DMR indicates that the methylation level is higher in ‘Chenghong Dangui’ than in ‘Zaohuang’. B, Difference in CpG methylation level. C–E, Genomic distribution of CG, CHG, and CHH sites in hyper DMRs in ‘Zaohuang’. F–H, Genomic distribution of CG, CHG and CHH sites in hypo DMRs in ‘Zaohuang’.

Differentially methylated region (DMR) analysis revealed significant differences in the distribution of hyper-DMRs and hypo-DMRs in the 23 chromosomes of ‘Zaohuang’ relative to ‘Chenghong Dangui’ (Fig. 2A), with some regions in candidate DMR regions being stable and some regions being hypermethylated or reduced in methylation; 13 372 DMR regions were higher in ‘Zaohuang’ than in ‘Chenghong Dangui’ (hyper DMR), and 10 491 were lower in ‘Zaohuang’ than in ‘Chenghong Dangui’ (hypo-DMR) (Fig. 2B, Supplementary File 1). These DMR regions may be associated with the expression of specific genes and also suggest that there may be large differences in gene expression between ‘Zaohuang’ and ‘Chenghong Dangui’ petals. In the hyper-DMRs of ‘Zaohuang’ relative to ‘Chenghong Dangui’, 51.25% of CG sites, 34.99% of CHG sites, and 36.95% of CHH sites occurred within <3000 bp of the gene promoter region (Fig. 2C–E). In the hypo-DMRs, 52.42% of CG sites, 35.36% of CHG sites, and 41.83% of CHH sites occurred within <3 kb of the gene promoter region (Fig. 2F–H). In addition, the promoter region was the region with the highest abundance of DMRs. Results showed that hyper-DMRs and hypo-DMRs of the sweet osmanthus genome were mainly distributed in gene promoter regions, indicating that methylation plays an important role in the regulation of gene expression in sweet osmanthus. The methylation ratio of the CHH sites in the promoter regions of ‘Chenghong Dangui’ was much higher than that in ‘Zaohuang’. CHH methylation is associated with RNA-directed DNA methylation (RdDM) [33, 34]. This implies that more genes may undergo post-transcriptional silencing and that gene expression may be more susceptible to regulation by post-transcriptional silencing in the petals of ‘Chenghong Dangui’.

### Validation of ATAC-seq datasets from petals of sweet osmanthus cultivars ‘Zaohuang’ and ‘Chenghong Dangui’

An average of more than 86 million reads were obtained for the ‘Chenghong Dangui’ triplicates, and an average of 94 million reads were obtained for the ‘Zaohuang’ triplicates through paired-end sequencing (Supplementary Table 4. By sequence alignment to the ‘Rixiang Gui’ genome, we found that an average of 85% of the ‘Chenghong Dangui’ reads and 84% of the ‘Zaohuang’ reads mapped to the ‘Rixiang Gui’ genome (Supplementary Table 5). More than 18 million reads per replicate passed the quality filtering stage of analysis (Supplementary Table 6), which is more than sufficient to successfully identify accessible chromatin regions in *Arabidopsis* [35]. The processed alignment files were used for comparative analysis of the six libraries using principal component analysis, and variation was greater between the ‘Chenghong Dangui’ and ‘Zaohuang’ samples, with low variation between the replicate samples, indicating a high level of reproducibility in our datasets (Supplementary Fig. 2).

**Figure 3:**
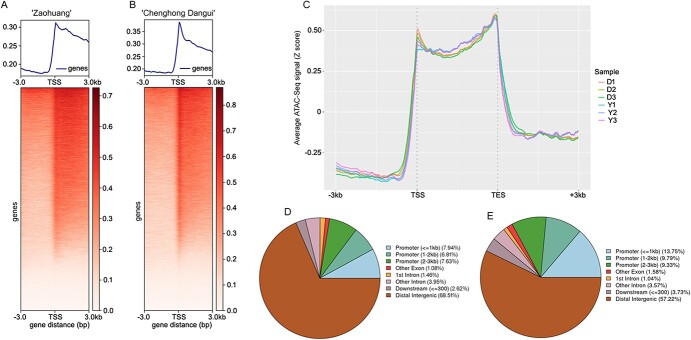
ATAC-seq data analysis of sweet osmanthus ‘Zaohuang’ and ‘Chenghong Dangui’ petals. A, TSS enrichment of sweet osmanthus ‘Zaohuang’. B, TSS enrichment of sweet osmanthus ‘Chenghong Dangui’. C, Distribution of ATAC-seq signal in gene body. D, Distribution of DAR gain in ‘Chenghong Dangui’. E, Distribution of DAR gain in ‘Zaohuang’. Y, ‘Zaohuang’; D, ‘Chenghong Dangui’.

ATAC-seq sequencing results showed that transcription start site (TSS) enrichment occurred in both ‘Zaohuang’ and ‘Chenghong Dangui’ cultivars, and the TSS enrichment signal was stronger in ‘Chenghong Dangui’ than in ‘Zaohuang’ (Fig. 3A–C). The average number of genes exhibiting TSS enrichment in ‘Chenghong Dangui’ was 29 728, and the average number of genes showing TSS enrichment in ‘Zaohuang’ was 28 843 (Supplementary File 2). A maximum peak of enriched genes was reached in the TSS region, and the peak of enriched genes decreased upstream or downstream of this region (Fig. 3A–C). This result indicated that the degree of chromatin accessibility was closely related to gene expression. An average of 130 067 and 130 096 total peaks were detected in petals of ‘Chenghong Dangui’ and ‘Zaohuang’, respectively. Irreproducible discovery rate (IDR) peak analysis revealed an average of 26 211 IDR peaks in ‘Chenghong Dangui’ and 21 741 IDR peaks in ‘Zaohuang’ replicate samples (Supplementary Table 6). Differentially accessible regions (DARs), were enhanced, with 6106 and 6080 in ‘Chenghong Dangui’ and ‘Zaohuang’, respectively (Supplementary File 3). The statistical analysis of DAR distribution revealed that the DAR gain and loss in the ‘Zaohuang’-vs-‘Chenghong Dangui’ comparison were mainly distributed in distal intergenic and promoter regions (<3 kb). The distribution ratios of DAR gains in these two regions in ‘Chenghong Dangui’ were 68.51% and 22.38%, respectively, and the distribution ratios of DAR loss in these two regions were 57.22% and 32.87% (Fig. 3D–E). Distal intergenic regions represent areas of mostly unknown function, which may be related to the remote regulation of genes. Except for distal intergenic regions, both DAR gain and DAR loss were identified in promoter regions, and there were more DAR gain regions in the promoter regions of ‘Zaohuang’ than in those of ‘Chenghong Dangui’. These results indicated that promoter regions in the ‘Zaohuang’ genome are more accessible than those in the ‘Chenghong Dangui’ genome, and there should be greater gene upregulation in the petals of ‘Zaohuang’.

### Reduced methylation and increased chromatin accessibility lead to upregulation of linalool metabolic pathway-related genes in ‘Chenghong Dangui’ petals

**Figure 4:**
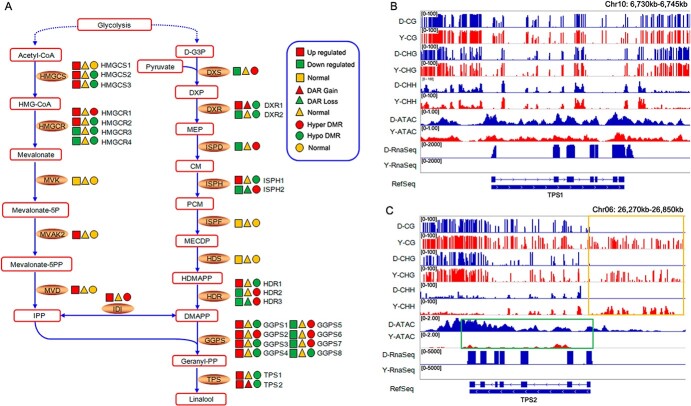
Effects of methylation level and chromatin accessibility on the expression of genes related to linalool production in sweet osmanthus ‘Chenghong Dangui’ petals (‘Zaohuang’-vs-‘Chenghong Dangui’). A, Methylation level and chromatin accessibility analysis of related genes in linalool production. D-G3P: D-glyceraldehyde 3-phophate, DXP: 1-deoxy-D-xylulose  5-phosphate, MEP: 2-C-methyl-D-erythritol 4-phosphate, CM: 4-(cytidine 5′-diphospho)-2-C-methyl-D-erythritol, PCM: 2-phospho-4-(cytidine 5′-diphospho)-2-C-methyl-D-erythritol, MECDP: 2-C-methyl-D-erythritol 2,4-cyclodiphosphate, HDMAPP: 1-hydroxy-2-methyl-2-butenyl 4-diphosphate, DMAPP: dimethylallyl-PP, HMG-CoA: 3-hydroxy-3-methyl-glutaryl-CoA, IPP: isopentenyl-PP, DXS: 1-deoxy-D-xylulose-5-phosphate synthase, DXR: 1-deoxy-D-xylulose-5-phosphate reductoisomerase, ISPD: 2-C-methyl-D-erythritol 4-phosphate cytidylyltransferase, ISPH: 4-hydroxy-3-methylbut-2-enyl diphosphate reductase, ISPE: 4-diphosphocytidyl-2-C-methyl-D-erythritol kinase, ISPF: 2-C-methyl-D-erythritol 2,4-cyclodiphosphate synthase, HDS: 1-hydroxy-2-methyl-2-(E)-butenyl-4-diphosphate synthase, HDR: 1-hydroxy-2-methyl-2-(E)-butenyl-4-diphosphate reductase, GGPS: geranylgeranyl pyrophosphate synthase, HMGCS: hydroxymethyl glutaryl-CoA synthase, HMGCR: hydroxymethyl glutaryl-CoA reductase, MVK: mevalonate kinase, MVAK2: phosphomevalonate kinase, MVD: diphosphomevalonate decarboxylase, IDI: isopentenyl diphosphate isomerase, TPS: terpene synthase. B–C, DNA methylation level and chromatin accessibility analysis of *TPS1* and *TPS2* genes. DMRs upstream of *TPS2* in ‘Chenghong Dangui’ and ‘Zaohuang’ are marked with a yellow box. DARs of *TPS2* in ‘Chenghong Dangui’ and ‘Zaohuang’ are marked with a green box. Y, ‘Zaohuang’; D, ‘Chenghong Dangui’.

Analysis of the expression of genes involved in the linalool production pathway showed that multiple genes were upregulated in ‘Chenghong Dangui’ petals: three *HMGCSs*, two *HMGCRs*, *MVAK2*, *MVD*, *DXR*, *ISPH*, *HDR*, *IDI*, four *GGPSs*, *TPS1*, and *TPS2* (Fig. 4A, Supplementary Fig. 3). The upregulation of these genes promoted metabolic flow toward linalool production and the accumulation of linalool in the petals of ‘Chenghong Dangui’, which was the main reason for its higher petal linalool content compared with ‘Zaohuang’. Among genes with upregulated expression, the methylation levels of *HMGCS2*, *HMGCS3, HMGCR2, ISPH1, HDR1, GGPS1, GGPS3, GGPS4*, and *TPS1* were reduced in ‘Chenghong Dangui’ petals, indicating that the expression of these genes was regulated by methylation. Both *DXR1* and *TPS2* genes had increased chromatin accessibility and decreased methylation levels in ‘Chenghong Dangui’ petals, whereas chromatin accessibility was decreased and methylation was increased in ‘Zaohuang’ petals (Fig. 4A).

TPS1 and TPS2 have been shown to catalyze the synthesis of S-linalool from the substrate GPP (geranyl pyrophosphate) [2]. The average methylation ratios of CG, CHG, and CHH in the 3-kb region upstream of *TPS1* were 84.27%, 62.74%, and 14.86%, respectively, in ‘Zaohuang’ petals and 82.88%, 56.75%, and 15.60% in ‘Chenghong Dangui’ petals. The average methylation ratios of CG, CHG, and CHH in the 3-kb region upstream of *TPS2* were 51.50%, 42.63%, and 14.32%, respectively, in ‘Zaohuang’ petals, whereas no methylation was detected at CG, CHG, and CHH sites in the 3-kb region upstream of *TPS2* in ‘Chenghong Dangui’ petals. The methylation ratio of the 3-kb region upstream of both *TPS1* and *TPS2* was higher in ‘Zaohuang’ petals than in ‘Chenghong Dangui’ petals, especially for *TPS2*, in which the degree of methylation in the promoter region was much higher in ‘Zaohuang’ (Fig. 4A–C). The high methylation ratio of the promoter regions of *TPS1* and *TPS2* in ‘Zaohuang’ petals may inhibit the expression of these two genes in ‘Zaohuang’ petals.

The results of ATAC-seq analysis showed that chromatin accessibility of the promoter region of *TPS1* did not differ between ‘Chenghong Dangui’ and ‘Zaohuang’ petals, but the chromatin accessibility of the promoter region of *TPS2* was much higher in ‘Chenghong Dangui’ petals (Fig. 4B–C), and this increased chromatin accessibility was more favorable for the binding of transcription factors and thus promoted gene expression in ‘Chenghong Dangui’ petals.

As observed in the transcriptome analysis, the expression of *TPS1* and *TPS2* was 4.96- and 15.46-fold higher in the petals of ‘Chenghong Dangui’ than in those of ‘Zaohuang’ (Supplementary Fig. 3); this finding was consistent with the results of methylation and chromatin accessibility analyses. *TPS1* gene expression in the petals of ‘Zaohuang’ may be affected by DNA methylation in the promoter region, whereas *TPS2* gene expression may be affected by both promoter region DNA methylation and chromatin accessibility.

### Transcriptional regulation of *TPS2* gene expression

**Figure 5:**
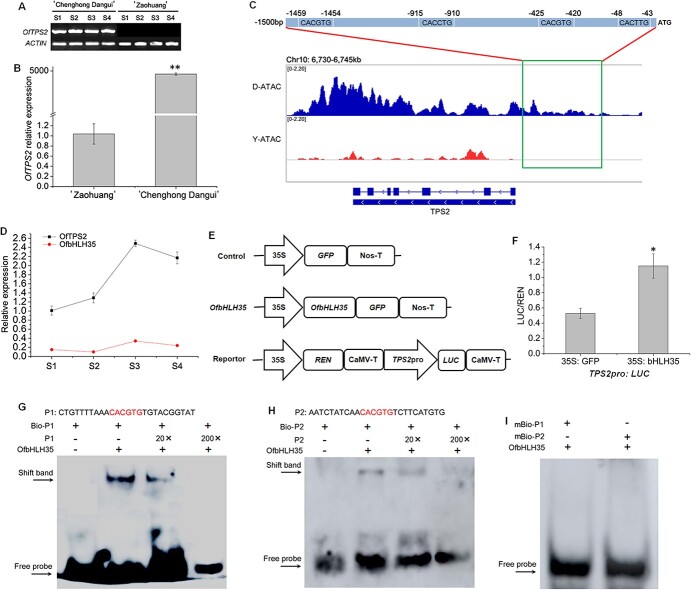
Expression and transcriptional regulation of *TPS2*. A, RT-PCR analysis of *TPS2* in sweet osmanthus petals at different stages. B, qRT-PCR analysis of *TPS2* in ‘Zaohuang’ and ‘Chenghong Dangui’ petals at the full flowering stage. The data represent the means ± SD of three replicates from three independent experiments. *P < 0.05, **P < 0.01. C, Chromatin accessibility upstream of the *TPS2* gene promoter in ‘Zaohuang’ and ‘Chenghong Dangui’. Y, ‘Zaohuang’; D, ‘Chenghong Dangui’. D, Expression patterns of *TPS2* and *bHLH35* in ‘Chenghong Dangui’ petals. E, The effector and reporter plasmids used in dual-LUC assays. REN, *Renilla* luciferase; LUC, firefly luciferase. F, *TPS2* promoter activity (LUC/REN ratio) of tobacco leaves co-infiltrated with *Agrobacteria* carrying effector and reporter. G–H, Binding activity of bHLH35 to the G-box elements. Competitors were added in 20- and 200-fold molar excess. I, Binding activity of bHLH35 to mutated probes.

Based on the transcriptome sequencing results, the fragments per kilobase of exon model per million mapped fragments (FPKM) value of *TPS2* was much higher than that of other genes in the metabolic pathway in ‘Chenghong Dangui’ petals compared with ‘Zaohuang’ petals (Supplementary Fig. 3). *TPS2* is a key gene for linalool synthesis. Quantitative real-time PCR (qRT-PCR) analysis showed that the gene was barely expressed in ‘Zaohuang’ petals and showed a 4642-fold increase in expression in ‘Chenghong Dangui’ petals (Fig. 5A–B). Chromatin accessibility analysis upstream of the *TPS2* gene promoter revealed the presence of an open region of approximately 1500 bp upstream of the start codon in ‘Chenghong Dangui’ petals but not in ‘Zaohuang’ petals. Sequence analysis revealed the presence of multiple bHLH transcription factor binding elements in this region (Fig. 5C, Supplementary Fig. 4), suggesting that *TPS2* may be regulated by bHLH transcription factors. Transcriptome sequencing analysis revealed that the transcription factor with the highest FPKM value among the transcription factors upregulated in ‘Chenghong Dangui’ petals was *bHLH35* (Supplementary Fig. 5A), which contained an open reading frame of 726 bp and encoded a protein with 242 amino acids (GenBank accession no. OM807065) (Supplementary Fig. 5B). qRT-PCR analysis showed that the expression patterns of *OfbHLH35* and *TPS2* in the petals of ‘Chenghong Dangui’ were similar at different time points (Fig. 5D). We used dual-LUC assays to investigate whether OfbHLH35 directly affected *TPS2* expression. When OfbHLH35 was expressed in *Nicotiana benthamiana* leaf cells harboring the TPS2pro: LUC plasmid, the promoter activity of *TPS2* significantly increased 2.17-fold compared with the control (Fig. 5E–F). Electrophoretic mobility shift assay (EMSA) analysis showed that the bHLH35 transcription factor could bind to the G-box elements upstream of *TPS2* (Fig. 5G–H). No binding signals were detected after the addition of the mutated probes (Fig. 5I). These results suggest that bHLH35 may directly bind to the G-box elements upstream of the *TPS2* promoter, thereby positively regulating its expression. In addition, among the transcription factors upregulated in the petals of ‘Chenghong Dangui’, several bHLH, AP2/ERF, and TCP transcription factors were included. These may also be involved in the regulation of *TPS2* gene expression, modulating the production of linalool and linalool oxides, which are important aromatic components in the petals of ‘Chenghong Dangui’.

### Regulation of gene expression related to the production of ionone in sweet osmanthus

**Figure 6:**
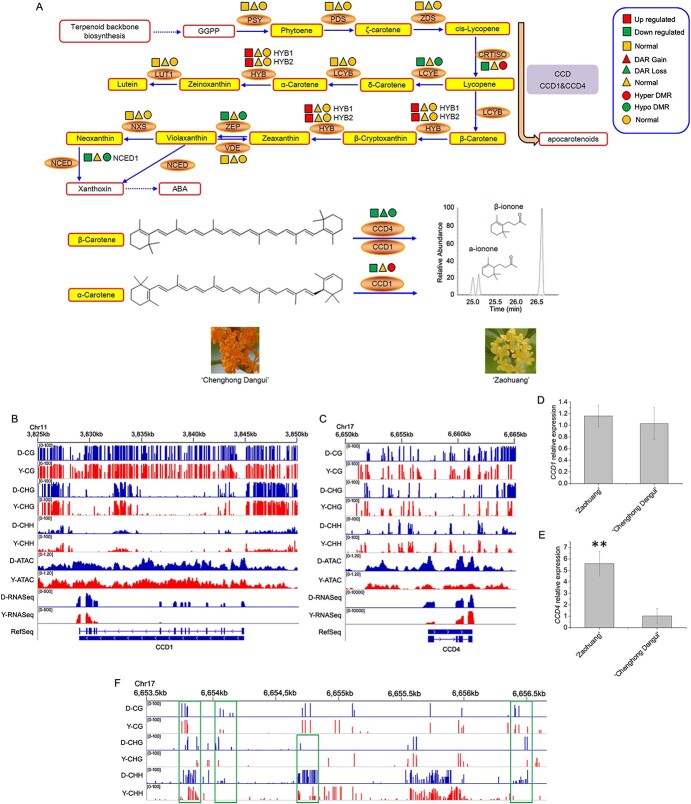
Effects of methylation level and chromatin accessibility on the expression of genes related to ionone production in sweet osmanthus ‘Chenghong Dangui’ petals (‘Zaohuang’-vs-‘Chenghong Dangui’). A, Methylation level and chromatin accessibility analysis of related genes in ionone production. GGPP: geranylgeranyl diphosphate, PSY: phytoene synthase, PDS: phytoene desaturase, ZDS: ζ-carotene desaturase, CRTISO: carotene isomerase, LCYB: lycopene β-cyclase, LCYE: lycopene ε-cyclase, HYB: β-carotene hydroxylase, LUT1: carotene ε-monooxygenase, ZEP: zeaxanthin epoxidase, VDE: violaxanthin de-epoxidase, NXS: neoxanthin synthase, NCED1: 9-cis-epoxycarotenoid dioxygenase, ABA: abscisic acid, CCD1: carotenoid cleavage dioxygenase 1, CCD4: carotenoid cleavage dioxygenase 4. B–C, DNA methylation level and chromatin accessibility analysis of *CCD1* and *CCD4*. D–E, qRT-PCR analysis of *CCD1* and *CCD4* in petals of the two cultivars at the full flowering stage. F, DNA methylation level 3 kb upstream of *CCD4*. Higher methylation level regions 3 kb upstream of *CCD4* in ‘Chenghong Dangui’ are marked with green boxes. Y, ‘Zaohuang’; D, ‘Chenghong Dangui’.

Analysis of the expression and regulation of genes related to the ionone synthesis pathway in sweet osmanthus showed that the expression of *PSY*, *PDS*, *ZDS*, *LCYB*, *VDE*, and *NXS* in the ionone synthesis pathway did not differ between ‘Zaohuang’ and ‘Chenghong Dangui’ petals, nor did chromatin accessibility of the region where the genes are located or the methylation ratio 3-kb upstream of these genes (Fig. 6A). These results indicated that none of these genes are key to the regulation of ionone synthesis. The expression of *ZEP*, *NCED1*, *CCD1*, and *CCD4* genes in the metabolic pathway was downregulated in ‘Chenghong Dangui’ petals relative to ‘Zaohuang’, and downregulated expression of these genes may promote the accumulation of carotenoids, especially β-carotene, in ‘Chenghong Dangui’ petals, consistent with our previous findings that ‘Chenghong Dangui’ petals accumulate high levels of carotenoids [14]. The methylation ratios of the promoter regions of the *ZEP* and *NCED1* genes were lower in ‘Chenghong Dangui’ than in ‘Zaohuang’, and chromatin accessibility did not differ between the two cultivars. However, *ZEP* and *NCED1* showed 1.29- and 3.28-fold decreases, respectively (Supplementary Fig. 6), in expression in ‘Chenghong Dangui’, indicating that the increased methylation levels of the promoter regions of these two genes in ‘Zaohuang’ petals did not affect gene expression, and the expression of these two genes may be regulated mainly by transcription factors.

*CCD1* and *CCD4* genes are important for the production of ionone by carotenoid cleavage in sweet osmanthus. *CCD1* and *CCD4* are highly expressed in ‘Zaohuang’ petals, and carotenoids, especially β-carotene, are cleaved to produce β-ionone; ‘Zaohuang’ petal essential oils therefore contain more ionone, but the flower color is lighter and pale yellow. By contrast, these two genes are expressed at low levels in ‘Chenghong Dangui’ petals, which accumulate a large amount of carotenoids, and ‘Chenghong Dangui’ petal essential oils therefore contain less ionone, but the cultivar has an orange-red flower color (Fig. 6A). *CCD1* and *CCD4* genes are key genes that regulate flower color and ionone synthesis in sweet osmanthus.

ATAC-seq analysis showed that chromatin accessibility of *CCD1* in ‘Chenghong Dangui’ petals was not significantly different from that of ‘Zaohuang’ (Fig. 6B). Methylation analysis of the 3 kb of sequence upstream of *CCD1* showed that the methylation ratio in ‘Chenghong Dangui’ was not significantly different from that in ‘Zaohuang’. The average methylation ratios of CG, CHG, and CHH loci in ‘Chenghong Dangui’ were 87.78%, 83.73%, and 10.53%, respectively, whereas the average methylation ratios of these loci in ‘Zaohuang’ were 85.86%, 84.62%, and 11.08%. The mean methylation ratio of CG sites in the upper 3 kb of the promoter region of *CCD1* was slightly higher in ‘Chenghong Dangui’ than in ‘Zaohuang’, and the mean methylation ratios of CHG and CHH sites were slightly lower in ‘Chenghong Dangui’. qRT-PCR analysis showed that *CCD1* was slightly more expressed in petals of ‘Zaohuang’ than in those of ‘Chenghong Dangui’ (Fig. 6D). In addition, the FPKM values of *CCD1* in the two cultivars were much lower than those of *CCD4* in the petals of ‘Zaohuang’, indicating that the lower expression of the *CCD1* gene in the petals may be due to the higher methylation ratios of the CG and CHG sites in the upper 3 kb of the gene promoter region. Therefore, the *CCD1* gene may not be the major locus that affects carotenoid cleavage in sweet osmanthus petals.

The results of ATAC-seq analysis showed that the chromatin accessibility of *CCD4* was greater in ‘Chenghong Dangui’ petals than in ‘Zaohuang’ petals (Fig. 6C). At the same time, the average methylation ratios of CG, CHG, and CHH sites within 3 kb upstream of the *CCD4* promoter were 22.19%, 13.96%, and 11.75%, respectively, in ‘Chenghong Dangui’ and 25.92%, 18.58%, and 10.72% in ‘Zaohuang’. The average methylation ratios of CG and CHG sites in the ‘Chenghong Dangui’ genome were lower than those in the ‘Zaohuang’ genome, and the average methylation ratios of CHH sites were slightly higher in the ‘Chenghong Dangui’ genome (Fig. 6C). As observed in the qRT-PCR analysis, *CCD4* showed a 5.59-fold increase in expression in ‘Zaohuang’ petals (Fig. 6E). Further analysis of methylation within 3 kb upstream of the *CCD4* gene promoter in ‘Chenghong Dangui’ petals showed that there were several sites with methylation levels significantly higher than those in the *CCD4* gene promoter in ‘Zaohuang’ petals (Fig. 6F); these sites may be transcription factor binding sites, which are key sites for the regulation of *CCD4* gene expression. The upregulation of *CCD4* gene expression in ‘Zaohuang’ petals suggested that *CCD4* gene expression was not regulated mainly by chromatin accessibility and methylation regulation but may be primarily regulated by transcription factors.

### Sequence analysis of OfERF2 and expression profiling of *OfCCD4*, *OfCCD1*, and *OfERF2* genes

**Figure 7:**
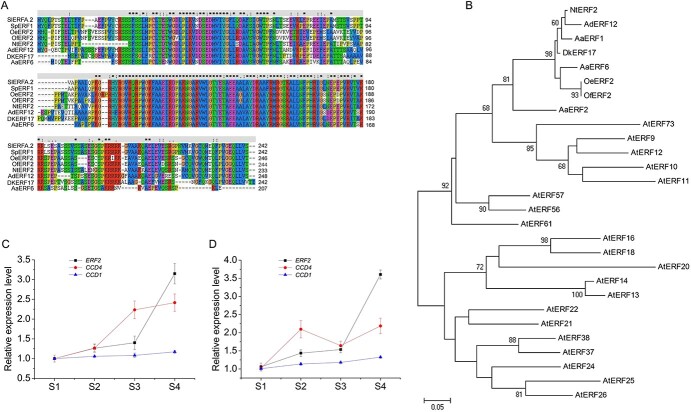
Sequence analysis of OfERF2 and expression profiling of *CCD4*, *CCD1*, and *ERF2* genes. A, The amino acid sequence of OfERF2 is aligned with the homologs *Solanum lycopersicum* ERFA.2 (SIERFA.2, NP_001316388.2), *Solanum pennellii* ERF1 (SpERF1, XP_015068116), *Olea europaea* ERF2 (OeERF2, XP_022890259), *Nicotiana tabacum* ERF2 (NtERF2, NP_001311965), *Actinidia deliciosa* ERF12 (AdERF12, ADJ67441), *Diospyros kaki* ERF17 (DKERF17, AID51422), and *Artemisia annua* ERF6 (AaERF6, AEQ93554). B, Phylogenetic relationships of OfERF2 with some other proteins. The accession numbers of AaERF2, AaERF6, AtERF9, AtERF10, AtERF11, AtERF12, AtERF13, AtERF14, AtERF16, AtERF18, AtERF20, AtERF21, AtERF22, AtERF24, AtERF25, AtERF26, AtERF37, AtERF38, AtERF56, AtERF57, AtERF61, and AtERF73 are AEQ93555, AGB07589, NP_199234, NP_171876, AEE30963, NP_174158, Q9CAP4, Q9LPE8, Q9C591, Q9S7L5, Q9C9I8, Q9C9I2, Q9LQ28, Q9SJR0, Q9FJ90, Q9CAN9, O80654, NP_181113, Q9SIE4, Q9FJQ2, Q9C7W2, and Q8H0T5. The analysis was performed by the NJ method using MEGA version 4.1. Bar, 0.2 substitutions per site. C–D, Expression profiles of *CCD4*, *CCD1*, and *ERF2* in sweet osmanthus ‘Zaohuang’ and ‘Chenghong Dangui’ flower petals.

ATAC-seq analysis revealed that the 3-kb region upstream of the *CCD4* gene promoter contains multiple chromatin-accessible regions that may be binding sites for transcription factors. Further sequence analysis revealed that these accessible regions contained multiple AP2/ERF transcription factor binding sites (Supplementary Fig. 7). Transcriptome sequencing analysis revealed a downregulated AP2/ERF homologous to *Nicotiana tabacum ERF2* named *OfERF2*. The cDNA of *OfERF2* (GenBank accession no. OM807064) was 732 bp in length and encoded 244 amino acids with a single AP2 domain. Sequence alignment showed that OfERF2 shared high amino acid sequence identity with *Olea europaea* ERF2, *N. tabacum* ERF2, *Artemisia annua* ERF6, *Diospyros kaki* ERF17, and *Actinidia deliciosa* ERF12 (Fig. 7A). The phylogenetic tree showed that OfERF2 had high similarity with OeERF2, DkERF17, AaERF1, and AaERF2 (Fig. 7B). AaERF1, AaERF2, and DkERF17 have been shown to participate in the regulation of plant secondary metabolism [36, 37]. Therefore, we speculated that OfERF2 may participate in the regulation of secondary metabolism, including terpenoid metabolism. The results of GWAS analysis of the genome and ornamental traits of sweet osmanthus showed that the *ERF2* gene is associated with flower color [38]. Moreover, qRT-PCR analysis showed that the expression pattern of *OfERF2* was consistent with that of *CCD4* and *CCD1* in petals of the two sweet osmanthus cultivars (Fig. 7C–D), suggesting that the OfERF2 transcription factor may participate in regulating the expression of *CCD4* and *CCD1* and is involved in regulating the cleavage of carotenoids, important pigments in sweet osmanthus flower color.

### Chromatin immunoprecipitation sequencing (ChIP-seq) analysis of genomic binding sites for the transcription factor OfERF2

**Figure 8:**
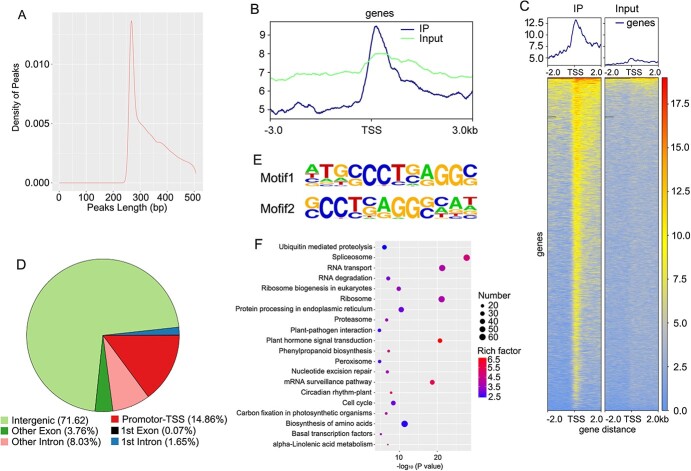
ChIP-seq analysis of the transcription factor ERF2. A, Length distribution of peaks. B–C, Distribution of reads on both sides of TSS. D, Distribution of peaks in genome. E, ERF2 binding motifs. F, KEGG enrichment results of peak-related genes.

ChIP-seq analysis showed that 2 953 490, 1 580 802, and 327 596 reads were obtained 10 kb, 5 kb, and 1 kb upstream of the TSS, respectively (Supplementary Table 7). Results of peak distribution analysis showed that the peak length was concentrated between 250–300 bp (Fig. 8A), and the distribution of reads on the genome was predominantly concentrated within 2 kb upstream and downstream of the TSS (Fig. 8B–C). The distribution of peaks in functional regions of the genome showed that, other than the intergenic region, the promoter region contained the most peaks, accounting for 14.86% (Fig. 8D). There were 6376 binding sites for the ERF2 transcription factor in TSS regions of different genes in the genome (Supplementary File 4). Among all the ERF2 binding sites, we found that most of the sequences contained motif1 and motif2, which contain the GCC core element (Fig. 8E). The KEGG enrichment results for peak-related genes showed that the three most enriched metabolic pathways were spliceosome, biosynthesis of amino acids, and ribosomes, with 62, 62, and 60 genes, respectively (Fig. 8F, Supplementary File 5). The terpenoid metabolic pathway had 17 enriched genes, the carotenoid metabolic pathway had 11 enriched genes, the phenylpropanoid metabolic pathway had 16 enriched genes, the flavonoid metabolic pathway had five enriched genes, and steroid biosynthesis had 11 enriched genes (Supplementary File 4). This indicates that the ERF2 transcription factor is widely involved in the regulation of secondary metabolism in plants.

**Figure 9:**
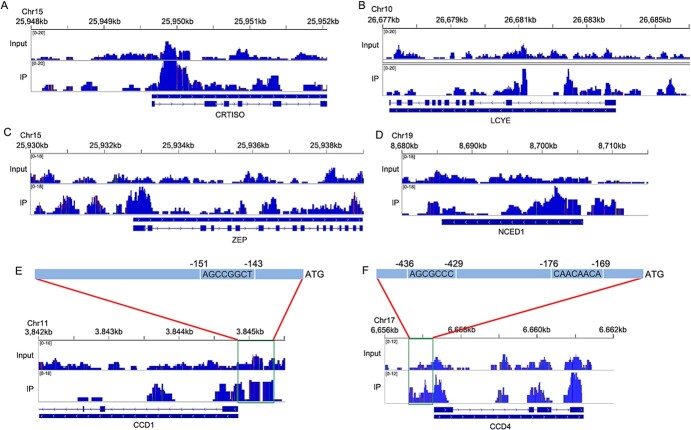
ChIP-seq peaks of upregulated genes in the carotenoid metabolic pathway. A, ChIP-seq peaks of *CRTISO*. B, ChIP-seq peaks of *LCYE*. C, ChIP-seq peaks of *ZEP*. D, ChIP-seq peaks of *NCED1*. E, ChIP-seq peaks of *CCD1*. F, ChIP-seq peaks of *CCD4*. Input was used as the control.

The results of peak analysis of *CRTISO*, *LCYE*, *ZEP*, *NCED1*, *CCD1*, and *CCD4*, which are upregulated in the carotenoid metabolic pathway in ‘Zaohuang’ petals, showed that the peak values of the promoter regions of *ZEP*, *NCED1*, *CCD1*, and *CCD4* genes were higher than those of the control (Fig. 9A–F), indicating that ERF2 can bind to their promoters to regulate their expression. *CCD4* and *CCD1* are key genes for carotenoid cleavage and ionone production in sweet osmanthus petals. We further analyzed the promoter regions of *CCD4* and *CCD1*. ChIP-seq results showed that there were significant peaks between 200–600 bp upstream of *CCD1* and between 100–500 bp upstream of *CCD4* (Fig. 9E–F), suggesting that these regions represent binding regions for the ERF2 transcription factor. Sequence analysis revealed the presence of a GCC element 151 bp upstream of the *CCD1* gene (Fig. 9E) and RAV1AAT and GCC-box elements 176 bp and 436 bp upstream of *CCD4* (Fig. 9F), all of which are binding sites for AP2/ERF transcription factors^4, 36, 39–40^. This suggested that ERF2 may regulate the expression of *CCD4* by binding to RAV1AAT and GCC-box elements and regulate the expression of *CCD1* by binding to the GCC-box element upstream of the genes*.*

### OfERF2 regulates the expression of *CCD1* and *CCD4* genes by binding to their promoter regions

**Figure 10:**
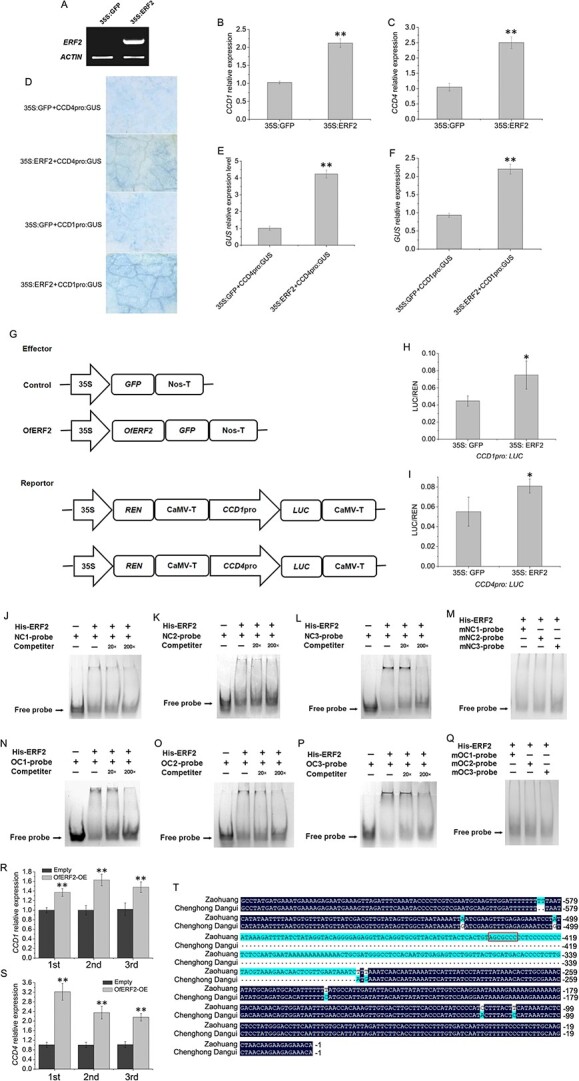
ERF2 promotes the transcription of *CCD1* and *CCD4* by binding to their promoters. A, RT-PCR detection of *ERF2* transcripts in tobacco leaves. B–C, qRT-PCR analysis of *NbCCD1* and *NbCCD4* transcript levels in control (35S:GFP) and transiently transformed tobacco leaves (35S:ERF2). D, *CCD1* and *CCD4* promoter activity in tobacco leaves co-infiltrated with *Agrobacteria* carrying the vectors. E–F, qRT-PCR analysis of *GUS* transcript levels in tobacco leaves after co-infiltration with *Agrobacteria* carrying the vectors. G, The effector and reporter plasmids used in dual-LUC assays. REN, *Renilla* luciferase; LUC, firefly luciferase. H–I, The *CCD1* and *CCD4* promoter activities (LUC/REN ratios) of tobacco leaves co-infiltrated with *Agrobacteria* carrying effectors and reporters. The data represent the means ± SD of three replicates from three independent experiments. *P < 0.05, **P < 0.01. J–L, Binding activity of ERF2 to the NC1, NC2, and NC3 probes. M, Binding activity of ERF2 to mutated NC1, NC2, and NC3 probes. N–P, Binding activity of ERF2 to the OC1, OC2, and OC3 probes. Competitors were added in 20- and 200-fold molar excess. Q, Binding activity of ERF2 to mutated OC1, OC2, and OC3 probes. R–S, qRT-PCR analysis of *NbCCD1* and *NbCCD4* transcript levels in control and sweet osmanthus ERF2 transgenic tobacco plants. T, Sequence alignment result of *CCD4* promoter regions in ‘Zaohuang’ and ‘Chenghong Dangui’.

Results of a transient transformation assay revealed an increase in *OfERF2* transcript levels in 35S:ERF2-containing *Agrobacterium tumefaciens-*infiltrated flowers compared with the control (Fig. 10A). The transcription of *N. benthamiana CCD1* (*NbCCD1*) and *NbCCD4* (https://solgenomics.net/) was affected by the overexpression of *OfERF2*, resulting in 2.10- and 2.50-fold increases, respectively, in expression in *OfERF2-*overexpressing petals compared with controls (Fig. 10B–C). When combinations of different vectors were transformed into tobacco leaves, the cultured and stained leaves appeared blue (Fig. 10D). By contrast, the leaf color of the control group was lighter, and that of tobacco leaves overexpressing *ERF2* was significantly darker (Fig. 10D). The qRT-PCR analysis indicated that when 35Spro:ERF2 and CCD4pro:GUS vectors and 35Spro:ERF2 and CCD1pro:GUS vectors were co-transformed into tobacco leaves, *GUS* expression was significantly upregulated (4.23- and 2.26-fold, respectively) compared with that of the control groups (Fig. 10E–F). We used dual-LUC assays to further investigate whether OfERF2 directly affected *CCD1* and *CCD4* expression. When *OfERF2* was expressed in *N. benthamiana* leaf cells containing the CCD1pro:LUC or CCD4pro:LUC plasmids, the promoter activities of *CCD1* and *CCD4* increased significantly 1.55- and 1.46-fold, respectively, compared with the control (Fig. 10G–I).

EMSAs were performed to test whether OfERF2 binds to the RAV1AAT and GCC-box elements in the *CCD1* and *CCD4* promoters. NC1 and NC2 from the *NbCCD1* promoter region, NC3 from the *NbCCD4* promoter region, OC1 from the *OfCCD1* promoter region, and OC2 and OC3 from the *OfCCD4* promoter region were used for the *in vitro* binding assays (Supplementary Table 8). Using EMSA, we observed bands representing free probes when the ERF2 protein was absent. A specific shifted band was observed when the NC1-labeled probe, NC2-labeled probe, NC3-labeled probe, OC1-labeled probe, OC2-labeled probe, and OC3-labeled probe were separately incubated with the ERF2 protein (Fig. 10J–L, N–P). Competition experiments were performed by adding competitors to the binding assay at 20- and 200-fold molar excesses. Binding signals were reduced upon the addition of a 20-fold unlabeled specific probe competitor and disappeared after the addition of 200-fold unlabeled probes (Fig. 10J–L, N–P). No binding signals were detected after the addition of NC1, NC2, NC3, OC1, OC2, or OC3 mutated probes (Fig. 10M, Q).

These results suggested that ERF2 acts as a transcription factor and directly binds to the AP2/ERF transcription factor-binding elements upstream of the *OfCCD1* and *OfCCD4* promoters, thereby positively regulating the expression of these genes. The results of qRT-PCR analysis showed that the expression of *NbCCD1* and *NbCCD4* was significantly upregulated in sweet osmanthus *OfERF2* transgenic tobacco flowers (Fig. 10R–S). These results showed that the OfERF2 transcription factor binds to functional elements in the promoters of the *CCD1* and *CCD4* genes to directly regulate their expression, thereby resulting in the synthesis of ionone, an important aromatic component of petals.

Sequencing results showed a deletion of 183 bp that was located 315 bp upstream of the *CCD4* start codon in the *CCD4* promoter region of ‘Chenghong Dangui’ but not of ‘Zaohuang’ (Fig. 10T). According to the results of the ChIP-seq analysis, the deletion coincided with the ERF2 transcription factor-binding region, and there was an ERF2 transcription factor-binding site (GCC-box) in the deleted sequence. Therefore, the low expression of *CCD4* in the petals of ‘Chenghong Dangui’ may be caused primarily by the absence of this functional element in the promoter region. This prevents the ERF2 transcription factor from binding, which in turn affects the expression of the *CCD4* gene.

## Discussion

Sweet osmanthus is a famous fragrant flowering plant in China. Among different sweet osmanthus cultivars, terpenoid aromatic components, including β-linalool, linalool derivatives, and β-ionone, are the main components of petal essential oils [5, 41, 42]. In the present study, we analyzed the petal aromatic components of 12 cultivars from four species groups of sweet osmanthus using GC–MS and found that all 12 contained high levels of linalool and its oxides. Most of the cultivars also contained high levels of β-ionone, with higher levels in the Albus group cultivars. In addition, some Luteus group cultivars contained high levels of α-ionone. This result also indicated that the monoterpene aromatic components linalool and its oxides and ionone are the main components of sweet osmanthus petal essential oils.

Gene expression is influenced by a combination of chromatin accessibility and the level of DNA methylation in the region where the gene is located. An open chromatin state facilitates the binding of transcription factors, thereby promoting gene expression [25, 43, 44]. The DNA methylation of genes affects transcription factor binding and gene transcription, thereby inhibiting gene expression and affecting plant growth and development [34, 45–47]. In the present study, the number of DAR gains in ‘Zaohuang’ petals and ‘Chenghong Dangui’ petals was 6106 and 6080, respectively, which were not significantly different. However, DAR gains involving the promoter region accounted for 22.38% in ‘Chenghong Dangui’ and 32.87% in ‘Zaohuang’. This indicated that the proportion of genes with promoter regions in the open state was much higher in ‘Zaohuang’ petals than in ‘Chenghong Dangui’ petals. The results of the methylation analysis showed that the methylation ratios of CG, CHG, and CHH sites in the gene promoter regions were higher in ‘Chenghong Dangui’ petals than in ‘Zaohuang’ petals. These results imply that there should be more downregulated genes in the petals of ‘Chenghong Dangui’. This might be influenced by the reduced accessibility of chromatin and upregulated DNA methylation levels in the gene promoter regions. Transcriptome sequencing results showed that 5820 genes were downregulated in ‘Chenghong Dangui’ petals and 4035 genes were downregulated in ‘Zaohuang’ petals in the ‘Zaohuang’-vs-‘Chenghong Dangui’ comparison (Supplementary File 6). This result was consistent with the results of chromatin accessibility and methylation analyses in the promoter regions of the two cultivars. These results indicate that increased chromatin accessibility in the promoter regions of genes and reduced methylation levels promoted the upregulation of more genes in ‘Zaohuang’ petals.

Promoter DNA methylation usually inhibits gene transcription. However, it promotes *ROS1* gene transcription in *A. thaliana* [48]. In tomatoes, hundreds of genes with increased DNA methylation were upregulated in *sldml2* mutants compared with the WT [29]. In this study, we also found that the expression levels of 1020 genes with promoter DNA hypermethylation in ‘Zaohuang’ were upregulated, as well as 512 genes with promoter DNA hypermethylation in ‘Chenghong Dangui’ (Supplementary Fig. 8). It is possible that DNA methylation inhibits the binding of transcriptional repressors of these genes [29].

Both the KEGG functional gene enrichment results of the DMR region and the differential peak genes in ATAC-seq showed that the MAPK signaling pathway, phenylpropanoid biosynthesis, and plant–pathogen interaction metabolic pathways were the most affected by methylation and chromatin accessibility (Supplementary Fig. 9). The MAPK signaling pathway is involved in plant growth, development, and abiotic and biotic stress response [49–54]. In sweet osmanthus petals, increased chromatin accessibility of multiple genes in the MAPK signaling pathway and plant-pathogen interactions promoted the expression of these genes and enhanced plant resistance to biotic and abiotic stresses during flowering. At the same time, the MAPK signaling pathway may interact with genes related to the phenylpropanoid metabolic pathway to regulate the synthesis of phenylpropanoid metabolites in sweet osmanthus petals.

In ‘Chenghong Dangui’ petals, no methylation sites were found within 5 kb upstream of the *TPS2* gene promoter, whereas the methylation ratios of CG, CHG, and CHH were 51.50%, 42.63%, and 14.32%, respectively, in ‘Zaohuang’ petals. At the same time, chromatin accessibility in the promoter region of *TPS2* was higher in ‘Chenghong Dangui’ petals than in ‘Zaohuang’ petals. Both the high methylation ratio in the promoter region and the reduced chromatin accessibility affected the binding of transcription factors to the promoter, thereby affecting gene expression, which may be the reason why the expression of *TPS2* was much higher in ‘Chenghong Dangui’ petals than in ‘Zaohuang’ petals. This may be the direct cause of the higher linalool content in ‘Chenghong Dangui’ petals than in ‘Zaohuang’ petals.

The bHLH transcription factor is extensively involved in the regulation of plant secondary metabolism. In tobacco, NtMYC2 regulates nicotine biosynthesis by binding directly to the G-box region of target gene promoters [55]. CrMYC2 acts as an early methyl jasmonate response factor by regulating *ORCA* gene expression and thereby regulating the expression of a series of terpenoid alkaloid synthase genes, including strictosidine synthase and tryptophan decarboxylase in *Catharanthus roseus* L. [56]. In tomato, bHLH1 acts as a negative regulator by binding to the promoters of *PSY, PDS*, and *ZDS* genes in the carotenoid metabolic pathway, thereby regulating carotenoid synthesis [57]. AtMYC2 is an important transcription factor in *A. thaliana* that directly and indirectly regulates the biosynthesis of active ingredients, and MYC2 activates expression by binding directly to the promoter regions of the sesquiterpene synthase genes *TPS21* and *TPS11* [58]. In the present study, the bHLH35 transcription factor could bind to the G-box element upstream of the *TPS2* gene promoter, suggesting that the bHLH35 transcription factor is involved in the regulation of *TPS2* gene expression. Expression of the *TPS2* gene should be regulated by chromosome accessibility, methylation of DNA in the promoter region, and transcription factors. The increased chromatin accessibility of the *TPS2* gene and the absence of methylation sites found in its promoter region in ‘Chenghong Dangui’ petals facilitate the binding of bHLH35 and other transcription factors to promote *TPS2* gene expression, resulting in the production of more linalool and linalool oxides in ‘Chenghong Dangui’ petals. After synthesis, these compounds may be transported to the cell surface in the form of glycosides and then hydrolyzed by glycosidase [59, 60]. Therefore, a small number of black granules of floral components could be seen in the cell walls of ‘Chenghong Dangui’ petal cells under cryo-scanning electron microscopy, and these black granules should be mainly aggregates of linalool and its oxides [4].

Sweet osmanthus CCD4 is involved in the production of flavor and aroma compounds by using different carotenoid substrates with variable specificity and cleavage sites [8, 9]. The protein encoded by *CCD4* can cleave β-carotene into β-ionone [9, 11]. Our previous study demonstrated that sweet osmanthus WRKY3 and ERF61 transcription factors can bind to the W-box and RAV1AAT elements, respectively, in the promoter of the *CCD4* gene to promote *CCD4* gene expression, and these two transcription factors may directly regulate the expression of *CCD4*, thereby regulating carotenoid cleavage and β-ionone production [4, 12]. Multiple AP2/ERF transcription factor binding sites were identified in the open region of chromatin upstream of the *CCD4* gene promoter, and the gene may also be regulated by AP2/ERF transcription factors. In the present study, ChIP-seq results for the OfERF2 transcription factor in sweet osmanthus showed that the peak value in the promoter region of the *CCD4* gene was significantly higher than that of the control, indicating that the OfERF2 transcription factor could bind to the promoter of *CCD4* to regulate its expression. *A. annua* ERF1 and ERF2 recognize RAV1AAT elements in the promoter region of target genes to activate their expression in the artemisinin biosynthesis pathway in *A. annua* [36]. *N. tabacum* ERF2 interacts with the GCC-box to positively or negatively modulate GCC-box-mediated gene expression [39, 40]. In this study, we demonstrated that the OfERF2 protein could interact with both the RAV1AAT and the GCC-box in the *CCD4* promoter and regulate its expression, thereby regulating the cleavage of carotenoids and the synthesis of β-ionone in sweet osmanthus petals. After its production, β-ionone may be translocated to the cell surface in a glycosylated form and secreted. Under cryo-scanning electron microscopy, a large number of black granules of floral components aggregated in the cell walls of ‘Zaohuang’ petals could be seen, and these black granules were mainly aggregates of β-ionone. The number of black particles was much higher on the cell walls of ‘Zaohuang’ petals than on those of ‘Chenghong Dangui’ petals, probably because the boiling point of β-ionone is higher than that of linalool and its oxides and its volatility is lower. Therefore, under freezing conditions, β-ionone is less volatile and more likely to aggregate into granules^4^.

In this study, we analyzed the genome-wide methylation status and chromatin accessibility of the sweet osmanthus cultivars ‘Zaohuang’ and ‘Chenghong Dangui’. We carried out an analysis of methylation levels involving genomic DNA, chromatin accessibility, and transcription factor regulation and their effect on the expression and modulation of linalool and ionone synthesis pathways. Differences in the content of linalool aromatic components in petals of the two sweet osmanthus cultivars were mainly regulated by DNA methylation and chromatin accessibility, whereas differences in the ionone content were mainly the result of transcription factor regulation. These results provide an important resource for further in-depth study of the regulatory mechanisms involving genome-wide gene expression during flower development in sweet osmanthus, and they provide an important theoretical basis for the utilization of floral fragrance components and the breeding of improved floral fragrance phenotypes in sweet osmanthus.

## Materials and methods

### Plant materials

Freshly cut flowering branches of *O. fragrans* ‘Zaohuang’ (Albus group) and ‘Chenghong Dangui’ (Aurantiacus group) were incubated at 22°C and 70% relative humidity under a 12 h/12 h light/dark cycle. Flowers were collected for analysis at the linggeng stage (S1), xiangyan stage (S2), initial flowering stage (S3), and full flowering stage (S4).

### GC–MS analysis of sweet osmanthus petals

Sweet osmanthus petals were sealed in 15-mL extraction flasks. After equilibration and headspace extraction, the extraction head was removed, and the sample was loaded into the GC–MS system. The chromatography and mass spectrometry conditions were as described in the method of Han et al. [4].

### RNA extraction and transcriptome sequencing

RNA sequencing (RNA-seq) was performed by Frasergen (Wuhan, China). Transcriptome datasets were generated using the Illumina HiSeq 2500 sequencing platform (San Diego, CA, USA). Gene expression levels of differentially expressed genes (DEGs) were computed using the following formula: FPKM = cDNA fragments / [mapped fragments (millions) × transcript length (kb)].

### Whole-genome bisulfite sequencing (WGBS)

WGBS was performed by Frasergen (Wuhan, China). Genomic DNA was fragmented to an appropriate size range (300–500 bp), and the ends of repaired DNA fragments were blunt-ended. After bisulfite treatment, the DNA was purified and recovered for PCR amplification. The amplified library was purified for quality inspection, and the qualified library was sequenced. Bismark software was used to compare the filtered data with the reference sequence [61], the dmrseq software package was used for analysis of differentially methylated regions [62], and the obtained DMR candidate regions were filtered. The filtering standard had a Q value of ≤0.05.

### ATAC-seq analysis

ATAC-seq was performed by Frasergen (Wuhan, China). The sucrose precipitation method was used in the experiment [63]. First, the petal tissue was collected, and a cell suspension was prepared. Cell membrane lysates were added to lyse the cells to obtain the nucleus. Then, Tn5 transposase was added to cut open DNA. The DNA fragments cut by the enzyme were amplified by PCR and then sequenced by second-generation high-throughput sequencing. Clean data were obtained after filtering the original sequencing data, and the clean data were compared with the reference genome sequence using Bowtie2 software [64]. The DARs of chromatin were analyzed with DESeq2 software [65]. The DAR filtering threshold was log_2_(fold change) ≥ 1 and *p*-value ≤ 0.01.

### ChIP-seq analysis

ChIP-seq was performed by Wuhan SeqHealth Tech Co., Ltd. (Wuhan, China). Sweet osmanthus petals at the full flowering stage were collected for the ChIP-seq experiment, which was performed using previously described methods [66, 67]. First, 0.1 g of flower petals were cross linked with 1% formaldehyde. The tissue cells were then broken, cytoplasmic contents were released, and nuclei were collected. The nuclei were lysed, the DNA protein-soluble complex was extracted, and DNA was broken into 200–1000 bp fragments. The original sequencing data were filtered to obtain high-quality clean reads and to compare the clean data with the reference genome of *O. fragrans*. The input was taken as the background, and MACS software was used for peak calling IP [68].

### Isolation and sequence analysis of OfERF2

Two primers, ERF2f and ERF2r (Supplementary Table 8), were synthesized to amplify the full-length *ERF2* gene. The PCR product was cloned into the pMD19-T vector and sequenced. We then compared the amino acid sequences of OfERF2 with those of ERFs from other plant species. Sequences were aligned using Clustal W 1.83. Phylogenetic analysis was performed using MEGA 4.1.

### RT-PCR and qRT-PCR analysis

RT-PCR and qRT-PCR primers are listed in Supplementary Table 8. The expression level of ACTIN was used as a reference. Quantitative real-time PCR was performed using a Roche LightCycler 480II detection system. Relative expression levels were calculated using the 2^−ΔΔCt^ method, and each analysis included 3–5 replicates.

### Transient transformation in tobacco leaves

*OfERF2* cDNA was amplified and cloned into the pHBT vector, and the promoter regions of *CCD4* (1.06 kb) and *CCD1* (1.82 kb) were inserted into the pCAMBIA1391 vector. The resulting vector was transformed into *A. tumefaciens* strain EHA105. Agroinfiltration was carried out following the method described by Han et al. [4]. Infiltrated plants were maintained in the laboratory under continuous lighting for 24 h. Subsequently, they were moved to a greenhouse and kept at 22°C with a 16 h/8 h light/dark cycle. GUS activity was detected according to the method described by Han et al. [4].

### Tobacco leaf disc transformation

Cut tobacco leaf discs were placed and cultured on culture medium for 24–48 h and soaked in *Agrobacterium* solution for 3–5 min. The infected leaf discs were inoculated on pre-culture medium and incubated in the dark at 25°C for 24 h. The medium was washed with carboxybenzylpenicillin for 10 min. After the surface liquid was absorbed with filter paper, the leaf discs were transferred to differentiation medium for culture; the medium was changed every two weeks. When the buds grew to 2 cm, they were transferred to rooting medium. After the roots were cultured for three weeks, the sterile seedlings were moved to soil and transferred to an artificial climate chamber for culture.

### Dual-luciferase reporter assay

The cDNA sequences of *OfbHLH35* and *OfERF2* were ligated into the pHBT vector (effectors). The promoter regions of *CCD4* (1.06 kb), *CCD1* (1.82 kb), and *TPS2* (1.53 kb) were inserted into the pGreenII 0800-Luc vector (reporters). The resulting vectors were transformed into *A. tumefaciens* strain EHA105. EHA105 strains containing effectors and reporters were cotransformed into 5–6-week-old *N. benthamiana* leaves. The infiltrated plants were maintained in the incubator under continuous lighting for 72 h at 23°C. The LUC and REN activities were analyzed using a dual-luciferase assay kit (Solarbio, China). LUC/REN ratios were used to represent the relative activities of the gene promoters.

### *In vitro* protein synthesis and electrophoretic mobility shift assay

cDNA sequences of *OfERF2* and *OfbHLH35* were amplified and cloned into the pET-30a-c vector and then transformed into *Escherichia coli* BL21 competent cells for protein expression. The protein expression, purification, and EMSA were performed according to the methods described by Han et al. [4]. For EMSA, 3′ biotin labeling sense oligonucleotides containing the G-box, RAV1AAT, GCC-box, or mutated probes and the corresponding antisense strand sequence were synthesized (Supplementary Table 8).

## Supplementary Material

Web_Material_uhac096

## Data Availability

All RNA-Seq, ATAC-seq, whole-genome bisulfite sequencing, and ChIP-seq data generated in this study have been submitted to the NCBI Sequence Read Archive under Bioproject accession PRJNA792064.
